# CSM Software: Continuous Symmetry and Chirality Measures for Quantitative Structural Analysis

**DOI:** 10.1021/acs.jcim.4c00609

**Published:** 2024-07-02

**Authors:** Inbal Tuvi-Arad, Yaffa Shalit, Gil Alon

**Affiliations:** † Department of Natural Sciences, The Open University of Israel, Raanana 4353701, Israel; § Department of Mathematics and Computer Science, The Open University of Israel, Raanana 4353701, Israel

## Abstract

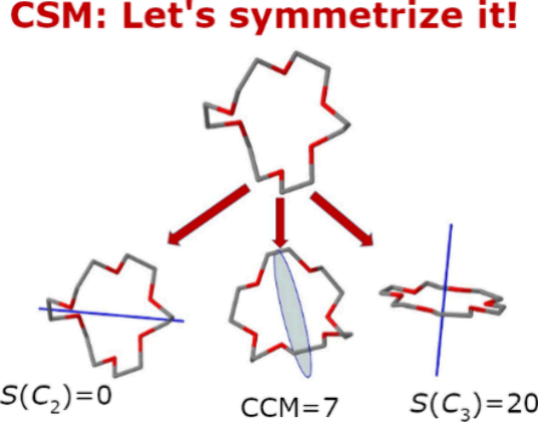

We present a comprehensive and updated Python-based open software to calculate continuous symmetry measures (CSMs) and their related continuous chirality measure (CCM) of molecules across chemistry. These descriptors are used to quantify distortion levels of molecular structures on a continuous scale and were proven insightful in numerous studies. The input information includes the coordinates of the molecular geometry and a desired cyclic symmetry point group (*i.e., C*_s_, *C*_i_, *C*_n_, or *S*_n_). The results include the coordinates of the nearest symmetric structure that belong to the desired symmetry point group, the permutation that defines the symmetry operation, the direction of the symmetry element in space, and a number, between zero and 100, representing the level of symmetry or chirality. Rather than treating symmetry as a binary property by which a structure is either symmetric or asymmetric, the CSM approach quantifies the level of gray between black and white and allows one to follow the course of change. The software can be downloaded from https://github.com/continuous-symmetry-measure/csm or used online at https://csm.ouproj.org.il.

## Introduction

Many molecules are naturally symmetric at their equilibrium state. Nevertheless, it is more likely to find them with near or approximate symmetry, as a result of instantaneous stretching, bending or twisting motion. Symmetry loss, or symmetry breaking, is not only a question of dynamics at very short time scales. It may result from conformational flexibility, substitution, reactive processes or phase transitions, under changing temperature and pressure as well as many other chemical and physical processes. Nonetheless, numerous experimental and computational studies show time and again that many of the special properties that emerge from perfect symmetry are still preserved in cases of near symmetry, only to a different extent. The goal of the CSM software is to quantify this “different extent”, on a continuous and normalized scale.

The acronym CSM stands for *Continuous Symmetry Measure* - a three-dimensional (3D) molecular descriptor that quantitatively estimates the distance of a molecule from its nearest symmetric structure with respect to a given symmetry point group *G*. This quantification converts the concept of symmetry from a binary yes/no property to a continuous one, and enables one to compare the 3D geometry of different structures on the same scale. As a continuous property, CSMs can be used to follow distortive processes and explore hidden insights about the mechanism of change. This concept opens the door to determine when symmetry serves as a driving force that controls the structure of molecules, or, alternatively, when does nature gives up on symmetry due to stronger driving forces (e.g., entropy), and if so, to what extent and why. In recent years we have revised and extended the algorithms of the CSM methodology, in order to increase the speed, accuracy and applicability of the method. Here we describe our revised open source software and its companion website, and provide guidelines for its usage.

The original development of the CSM methodology started at the early 90′s of the 20th century, with the seminal work of Zabrodsky, Peleg and Avnir.^1^ Following this paper, the method was extended to measure chirality^2^ and shape.^3^ In the following years, the method was applied to study the role of approximate symmetry in numerous molecular systems, including symmetry of crystals,^4,5^ distortion paths of inorganic complexes,^6^ reactive processes,^7−10^ host–guest interactions,^11^ molecular dynamics^12^ and symmetry of biomolecules.^13,14^ Application of this approach beyond chemistry were also documented, e.g., in archeology.^15,16^

The main challenge in calculating symmetry measures is to find the reference structure with the desired symmetry. This structure determines (and in fact, is also determined by) a direction vector of the generating symmetry operation and a permutation of the molecule’s atoms. Through the years several algorithmic strategies were used to tackle this challenge. The code described here is based on our recent algorithmic developments which we divide into three main approaches: 1) An exact algorithm for small-to-medium size molecules; 2) A set of approximate algorithms for large molecules; 3) An approximate algorithm for protein structures.

The original approach was based on a folding-unfolding algorithm introduced by Zabrodsky et al.^1^ This algorithm was later replaced with an analytical algorithm by Pinsky et al.^17^ Both of these algorithms scanned all mathematically possible permutations. Since the number of permutations grows very fast with the size of the molecule, such calculations are only feasible for small molecules. A third approach was presented in 2012 with an approximate algorithm to calculate the CSM of large molecular structures.^18^ In 2018 we introduced major algorithmic improvements and converted the code to python, due to its adaptability, simple syntax and robustness.^19^ The new algorithm replaced the mechanism of finding relevant permutations: Rather than scanning all the mathematically possible permutations the new code scans only chemically relevant permutations that maintain the connectivity of the molecule. It thus removed barriers related to the size of the molecules being studied, and opened the door for a huge variety of structures that previously required an approximate calculation. The same concept of structure preservation was applied to study the symmetry level of protein homomers, with an approximate CSM approach.^20^ In this version, we replaced the greedy algorithm originally used by Dryzun et al.^18^ with the Hungarian algorithm,^21^ in order to achieve better accuracy. Recently, several alternative algorithms to calculate the approximate CSM for general large structures were suggested by us.^22^ The CSM code described here is an up-to-date python version of the complete set of algorithms, spanning all types of molecules. The details of the various algorithms were extensively described in our previous methodological papers^19,20,22^ and will not be repeated. The focus in this paper is on the usability of the code.

## Execution Instructions

The CSM software is a command-line code that takes a molecular structure and the desired point group as input, and creates a directory with several output files based on the required calculation approach. The code uses Open Babel^23^ for the purpose of reading and writing molecules in various chemical formats. The different options are described below. Examples of the run commands and major options are provided as Supporting Information (SI).

### The Calculation Approach

Several calculation approaches are available.

1. Exact calculation (command = ″exact″) by which the code scans all possible permutations: either all the mathematically possible ones or only the chemically meaningful ones that preserve the essence of the chemical structure.
2. Approximate calculation (command = ″approx″) using the direction-permutation approach.
3. Trivial calculation (command = ″trivial″) in which a permutation search is not performed and the CSM is calculated using the identity permutation by which each atom is permuted with itself.
4. Combination of several calculations (command = ″comfile″) for the same set of molecules. These are specified in an additional input text file (default name = ″cmd.txt”).

Read, write and calculate options (commands = ″read″, ″write″, ″calculate″) are also available as separate commands in order to ease the integration of the code within other software.

### The Input Structure

The CSM code accepts molecular files in one of the following formats: mol, mol2, sdf, pdb, xyz, the Cambridge Structural Database (CSD) cor format and an internal csm format. Concatenated files of many molecular models, or a directory of molecular files are also acceptable input. Preferably one should use a format with connectivity data (e.g., mol, mol2, sdf, pdb, csm). For formats without connectivity, Open Babel^23^ is used to deduce it. Alternatively, an external connectivity file can be supplied by the user (see SI and Figures S1–S2 for specifications of the connectivity file and the csm format).

### Choosing the Point Group

The CSM is calculated with respect to a point group *G*, which is given by the user as part of the input. This point group characterizes the desired symmetry of the molecule. The current CSM software can handle the following point groups: *C*_s_, *C*_i_, *C*_n_ (n= 2, 3, 4, ...) and *S*_n_ (n = 4, 6, 8, ...). The CCM is calculated by setting *G = Ch*; In this case, the code calculates the CSM with respect to all achiral point groups (*S*_n_, n = 1, 2, 4, 6, ...); The reported CCM value is the minimal CSM. To speed up the calculation, the value of n in *S*_n_ can be limited with the flag sn-max.

### The Output Directory

By default, the program creates an output directory with several text files:

1. The applied command and version of the code (file name: ″version.txt″)
2. The resulting CSM or CCM values (file name: ″csm.txt″)
3. The final permutation (file name: ″permutation.txt″)
4. The direction vector of the symmetry element (file name: ″directional.txt″)
5. Two coordinate files: The original structure, for which the center of mass was translated to the origin (file name: ″initial_coordinates″); The resulting nearest symmetric structure (file name: ″resulting_symmetric_coordinates″). The format and extension of these files are determined by the format of the input molecule.

The program can also be operated with the ″--simple″ flag. In this case, there is no output directory, and the output reduces to the code version, some data about the equivalence groups and the CSM result, all are printed to the screen.

### Calculation Options

The CSM code offers many options that meet different analysis purposes. The main ones are described here along with their relevant code flags.

#### Options for Exact Calculation

1. keep-structure: This flag calls the main algorithm for scanning the structure preserving permutations. Exact calculation with this flag directs the code to scan permutations that maintain the original connectivity of the molecule, and skip all the others.^19^ In this way, the code looks for a reference symmetric structure that keeps the chemical essence of the original structure. In order to use this flag, connectivity data is needed. This can be part of the input coordinate file format, supplied by an additional connectivity file using the connect flag, or added by the babel-bond flag that directs the code to use Open Babel to determine the connectivity. Two comments are in order here:
  - When analyzing reactive processes in which bonds are breaking and new ones are created, it is advisible to avoid using the babel-bond flag and supply the code with a connectivity file that describes either the reactant, transition state or product connectivity in accordance with the relevant symmetry being formed or lost in the process. Doing so will direct the code to search for a more relevant symmetric structure when determining the reference structure.^24^
  - The code can operate without the keep-structure flag, and sometimes find a smaller CSM value in these cases, on the expense of breaking the connectivity map of the molecule when forming the nearest symmetric structure. This strategy can be used with small structures for which the total number of permutations can be scanned in a reasonable time frame.^22^
2. use-perm: As an alternative for permutation scanning, the user can supply a specific permutation as a text file using this flag. This is useful when a molecule displays approximate symmetry with respect to a noncyclic group with several symmetry elements of the same type, and the user is interested in analyzing the distortion with respect to a cyclic subgroup (e.g., *C*_s_ is a subgroup of any *C*_nv_ group). When the relevant permutation is the identity permutation, one can use the trivial approach (rather than exact) with no flags. In both cases the calculation is fast, and does not require connectivity data. The permutation file format is specified in the SI.
3. ignore-sym: With this flag the code ignores the chemical symbols of the atoms and treats all atoms as chemically equivalent. This option changes the CSM analysis into a mathematical shape descriptor. It is a slower calculation, suitable for small structures, unless a permutation is provided.
4. select-atoms: This flag allows the user to calculate the CSM for a fragment of the molecule, defined as a list of serial numbers provided by the user. This strategy is very powerful for analyzing sets of substituted molecules with a common symmetric core. Combined with the connect flag, the connectivity file should describe the connectivity of the original (complete) structure and not the fragment. In the output directory, only the fragment coordinates will be written (both for the input structure and the nearest symmetric structure), in which case, atoms’ indices will naturally change.
5. remove-hy: With this flag, hydrogen atoms are ignored in the symmetry analysis. In the output directory, the input structure and the nearest symmetric structure are written without the hydrogen atoms, and atoms’ indices change accordingly. This option is particularly useful for large structures when hydrogen atoms may considerably increase the number of permutations. Removing the hydrogen atoms often makes the exact calculation feasible even for very large molecules.
6. select-mols: This flag is useful for analyzing several models taken from a concatenated file with many molecules. The user needs to supply the indices of the relevant models in the file, and only these will be used in the symmetry calculation. Note that the code still reads the complete input file before extracting the relevant models and this may slow down the process.

#### Options for Approximate Calculation

The default approximate calculation uses the Hungarian algorithm^21^ and three initial direction vectors (along the Cartesian axes) to find the permutation of the atoms and, consequently, the direction of the symmetry element in space. Other algorithms are available with the following flags:

1. greedy: This flag calls for the greedy algorithm, as described by Dryzun et al.^18^ This is often the fastest calculation, but not necessarily the most accurate one in terms of structure preservation.^22^
2. keep-structure: Using this flag with the approx approach invokes the approximate structure preserving algorithm. This algorithm replaces the Hungarian algorithm^21^ with an algorithm that prioritize permutations with minimal distances between the permuted atoms.^22^
3. fibonacci n: This flag calls the Hungarian algorithm^21^ and apply the Fibonacci sphere algorithm^25^ to generate *n* initial direction vectors which are uniformly distributed on the unit sphere, as described in Alon et al.^22^ Note that n is an integer.

Several additional options are available for protein homomers in pdb format:

4. use-sequence: This flag uses the sequence data in the pdb file to determine which atoms are equivalent, based on their chain index, type of residue, serial number in the sequence as well as the chemical element.
5. use-chains: This flag invokes an algorithm that searches for the chains’ permutation along with the atoms’ permutation.
6. select-chains: This flag allows CSM calculation on selected chains of a protein oligomer.
7. select-res: This flag allows CSM calculation on selected residues of a protein oligomer. Residues are selected according to their serial numbers. The same set of residues is selected from all chains.
8. use-backbone: This option removes the atoms of the side-chains and uses only backbone atoms for the calculation.

As a simple alternative, the CSM of protein homomers can be calculated with the trivial approach, where the permutation of the atoms is predetermined according to their sequence numbers. When combined with the use-chain algorithm, the Hungarian algorithm is applied for finding the permutations between the chains, but the permutation of the atoms is still dictated by the sequence and is not searched.

It is important to state that pdb files may contain sequence gaps, alternative locations and other technical issues, that the CSM code does not handle. It is therefore required to clean and prepare pdb files for CSM calculations. Our python code pdb_prep(26) can be used for this purpose.^20^

## Online Calculation of Symmetry Measures

Given the coordinates of a molecule, the CSM and CCM can be calculated using the CoSyM website^27^ by uploading the molecular file and following the online menu. The website accepts a variety of molecular formats (xyz, mol, sdf, pdb, CSD’s cor files) as well as concatenated files with a list of molecules. The website was designed for both research and education purposes, with limited functionality as compared with the stand-alone software. It employs a time limit of up to 5 min on every run, and includes two calculators:

- The **molecule** calculator: CSM and CCM calculations with the exact approach for small-to-medium size molecules. An interface to calculate the continuous shape measure (CShM) using the original program developed by Pinsky and Avnir^3^ is also available, and is provided without modifications as a service to the scientific community. The CShM is derived from the CSM when the reference symmetric structure is a predefined shape, such as one of the regular polyhedral shapes (tetrahedron, octahedron, cube etc.). This is a separate program, and is not part of the revised CSM software.
- The **protein** calculator is designed for approximate symmetry analysis of protein homomers^20^ using the Hungarian or greedy algorithms as well as the trivial approach (i.e., a sequence-based permutation as explained above). Due to the time limit, only relatively small proteins can be processed. Protein files are accepted in pdb format or by their PDB-IDs as determined by the protein data bank.^28^ As discussed above, proteins should be cleaned prior to the CSM calculation. The website employs our python code pdb_prep(26) for this purpose.

In both calculators, a Jmol^29^ window is integrated in the interface in order to display the uploaded molecule or protein, as well as the nearest symmetric structure resulting from the calculation. The numerical results are displayed in a table that specifies the value of the measure (CSM, CCM), the type of calculation and its major parameters (e.g., the point group) and the direction of the symmetry element in space. This table as well as the coordinates of the structure can be downloaded. Further instructions regarding the usage of the website are available under the help tab on the website, and will not be repeated here.

## Usage Examples

Three examples for using the CSM software were selected in order to demonstrate the capabilities of the software. These include a flexible cage molecule for which the symmetry changes with the conformation or the guest, a crystal structure with pseudosymmetry of the unit cell and a protein that goes through a symmetry breaking process. The run commands, as well as input and output files for all the examples are provided as SI.

### Example 1: Flexible Cage Molecule

**Figure 1:**
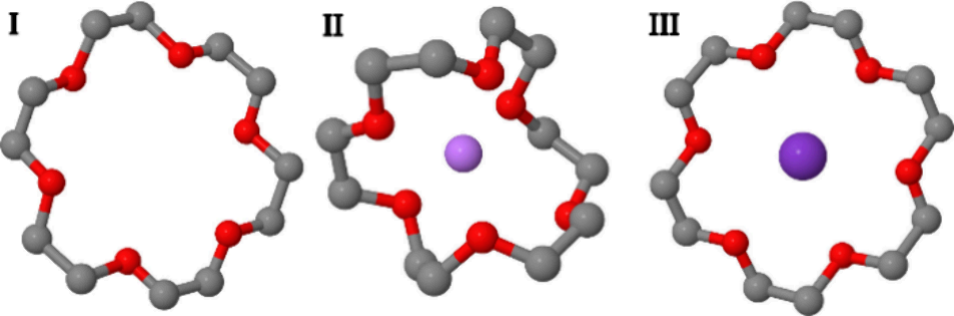
Crystal structures of 18C6 **I**. Isolated molecule: refcode = CENHIM, *S*(*C*_3_) = 0.0458, *S*(*C*_2_) = 0.000 **II**. 18C6 with Li^+^: refcode = FEDXUH, *S*(*C*_3_) = 18.0101, *S*(*C*_2_) = 0.3953. **III**. 18C6 with K^+^: refcode = AWEWOR, *S*(*C*_3_) = 0.0027 and *S*(*C*_2_) = 0.0072.

The 18-crown-6 molecule (18C6, C_18_O_6_H_36_) is a cage molecule with many applications in host–guest chemistry.^30^ It is commonly described as symmetric, but due to its flexibility, symmetry is not always conserved.^11^Figure 1 presents three models of the crystal structure of the molecule, extracted from the Cambridge Structural Database (CSD),^31^ and their CSM values with respect to the *C*_3_ and *C*_2_ point groups. CSM calculations were done with the exact approach using the keep-structure and remove-hy flags. Structure **I** is an isolated 18C6 (refcode = CENHIM) with perfect *C*_*2*_ symmetry (*S*(*C*_2_)=0.000) and slight distortion with respect to *C*_3_ (*S*(*C*_3_)=0.0458). Structure **II** is a complex of 18C6 with Li^+^ (refcode = FEDXUH), in which the host folds around the guest in order to increase nonbonding interactions. The distortion of the host with respect to *C*_3_ is high with *S*(*C*_3_) = 18.0101, while *S*(*C*_2_) = 0.3953 teaches on much smaller distortion with respect to *C*_2_. Replacing the Li^+^ ion with the much larger K^+^ ion (structure **III**, refcode = AWEWOR) forces symmetry on the host (*S*(*C*_3_)=0.0027 and *S*(*C*_2_)=0.0072) as the size of the guest perfectly matches the void space inside the host. In the gas phase, flexibility of this molecule translates to numerous conformations with different levels of distortion. CSM analysis of 18C6 complexes with Li^+^ and Na^+^ ions in the gas phase showed that distortion correlates with nonbonding interactions: higher distortion with respect to *C*_3_ is associated with stronger nonbonding interactions.^11^ This type of calculation is relatively fast. Using one core on our Intel(R) Xeon(R) Gold 6130 CPU@2.10 GHz Linux server, we calculated the CSM for the three molecules in Figure 1, and repeated the calculation five times. The average user time was 0.9 s per molecule and point group.

### Example 2: A Crystal with Pseudosymmetry

**Figure 2:**
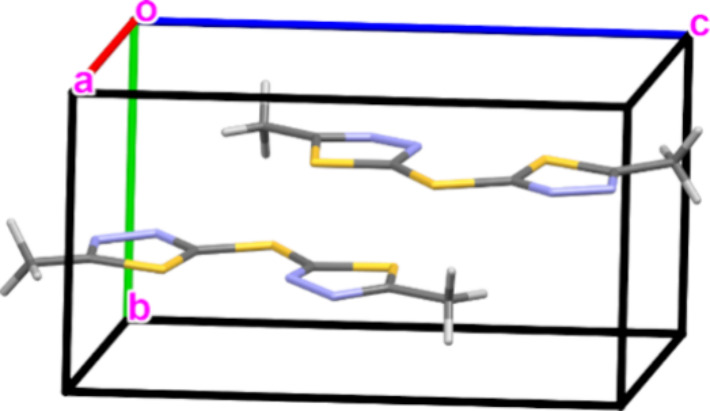
A crystal with pseudosymmetry of inversion. Refcode = CILHAI, *S*(*C*_i_) = 0.0140.

Figure 2 presents the unit cell of the crystal C_6_H_6_N_4_S_3_ (2,2′-Sulfanediylbis(5-methyl-1,3,4-thiadiazole), refcode = CILHAI) as extracted from the CSD. The crystal is considered asymmetric, with the space group P_1_. However, a CSM calculation reveals that its deviation from inversion symmetry is very small, *S*(*C*_i_) = 0.0140, teaching on higher pseudosymmetry. We note that there are two molecules in the unit cell (Z’=2). Calculation based on the exact approach with the keep-structure flag, using all the atoms that construct the unit cell, maintains the connectivity of the molecules as separate entities. The CSM value implies that a better description may be the P_–1_ space group. To time the calculation, we created 5 copies of the molecular file and calculated the CSM for all of them. We timed five repeats of this calculation, ending with an average user time of 0.6 s per crystal.

### Example 3: Conformational Change of the SARS-CoV-2 Spike Protein

**Figure 3:**
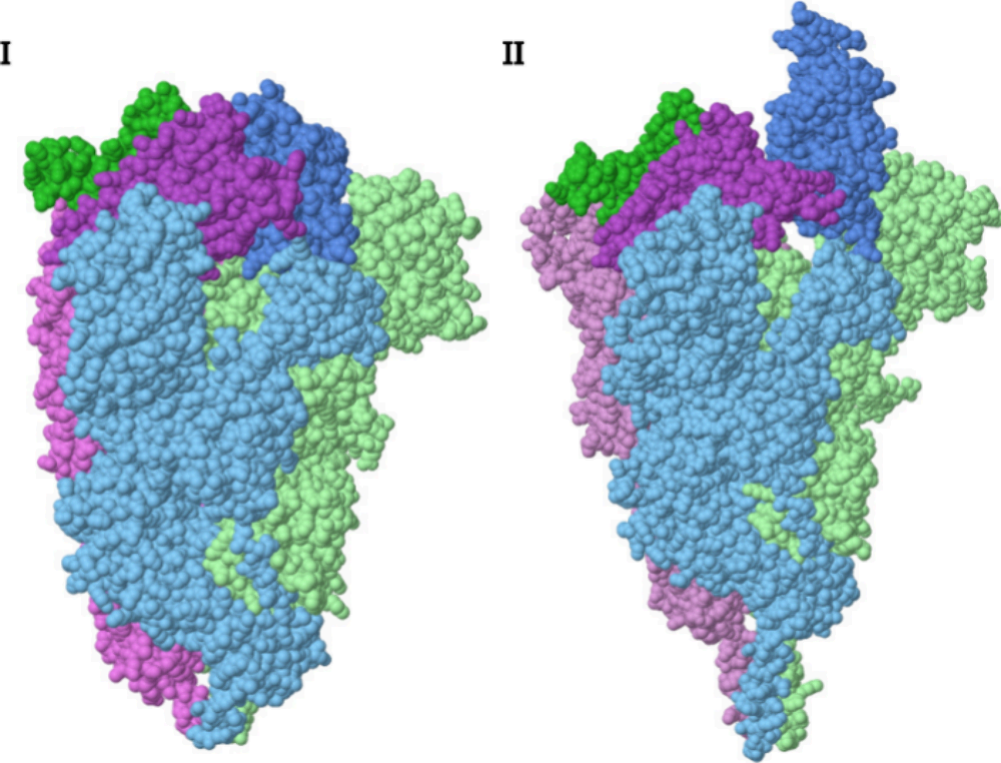
Two SARS-CoV-2 Omicron BA.1 spikes conformations. **I**. 3-Down (PDB: 7TF8). **II**. 1-Up (PDB: 7TO4). The color scheme is by chain: The migrating chain A in blue, chain B in green and chain C in purple. The RBD domains are in darker colors than the other domains to emphasize the conformational change.

The SARS-CoV-2 spike protein is a homotrimer glycoprotein directly involved in the infection process of the corona virus. A preliminary step of the infection process is a reversible migration of one chain of the receptor binding domain (RBD) that destroys the protein symmetry as illustrated in Figure 3. Interestingly, the conformational change has a negligible effect on the other domains of the protein. The CSM can be used to quantify this effect. Coordinates of Cryo-EM measurements of two representative spike proteins of the omicron BA.1 variant were retrieved from the RCSB-PDB,^28^ and are presented in Figure 3. The first (PDB-ID = 7TF8) is a symmetric 3-Down conformation and the second (PDB-ID = 7TO4) is an asymmetric 1-Up conformation. The pdb files were cleaned with our python script pdb_prep(26) to make sure that all chains are equal in length, without alternate location coordinates or noncoordinates lines. Calculations of *S*(*C*_3_) for the different subunits and domains of the spike protein were performed with the approx approach, applying the use-sequence, use-chain, and select-residues flags, including all the atoms of the side chains. The highest difference in distortion occurs at the RBD: *S*(*C*_3_) = 0.3910 (3-Down) and 12.3141 (1-UP). The other domains are mostly unaffected by the RBD migration (Table S2 in the SI presents additional details. Table S3 presents similar calculations at the backbone level applying the use-backbone flag). For estimating the software performance, we calculated the CSM of both proteins, repeated it five times, and averaged the results. On our Linux server the calculation took 64 s on average per protein when all atoms were included, 50 s when only backbone atoms were included, and 37 s for the RBD domain (including side chains). Additional performance data of the CSM software was published previously.^19,20,22^

## Summary

Symmetry is a fundamental property used to describe numerous types of molecules. Although it is often associated with minimum energy and stability, the common practice is that molecules are only approximately symmetric. The concepts of symmetry and chirality measures, first introduced by Zabrodsky et al.,^1,2^ provide a quantitative language to describe this level of approximation by a set of global 3D-descriptors of the geometry. Numerous studies showed through the years how symmetry and chirality measures explore hidden insights about molecular systems and often correlate with other physical and chemical properties such as temperature, pressure, reactivity and more. The revised CSM software described here allows fast and accurate calculation of these measures. An attractive feature of the method is its applicability to different types of molecules, including organic, inorganic and biochemical molecules at various sizes, from small molecules and up to macromolecules, biomolecules and large unit cells. We hope that the presented software and its companion website will expand the usage of this methodology in structural chemistry, and help reveal sources of distortion and mechanisms of change.

## Data Availability

CSM is available at https://github.com/continuous-symmetry-measure/csm. Online calculators with the main options are available at the CoSyM website: https://csm.ouproj.org.il. Input and output data files for the examples shown here are available at https://continuous-symmetry.github.io/CSM-OUI/Data.

## Supporting Information Available

Usage instruction, file formats, and additional tables (PDF)
Input, output, and readme files for examples included in the paper (ZIP)
