# Supplementary material for: CSM Software: Continuous Symmetry and Chirality Measures for Quantitative Structural Analysis
Source: J Chem Inf Model. 2024 Jul 2;64(14):5375–80. doi: 10.1021/acs.jcim.4c00609 (PMC11267602; doi:10.1021/acs.jcim.4c00609)
Supplement: Supplementary file 1 — ci4c00609_si_001.pdf [file ci4c00609_si_001.pdf]

Supporting Information

CSM Software: Continuous Symmetry and Chirality Measures for quantitative Structural Analysis

Inbal Tuvi-Arad[1*], Yaffa Shalit[1] and Gil Alon[2]

1. Department of Natural Sciences, The Open University of Israel, Raanana, Israel
2. Department of Mathematics and Computer Science, The Open University of Israel, Raanana, Israel

Contents

Connectivity File Format 1

Figure S1: Connectivity file example. 2

The `csm` File Format 2

Figure S2: The csm file format example 2

Permutation File Format 3

Table S1: Permutation file format examples. 3

Usage Examples – Run Commands 4

Example 1 – Flexible cage molecule 4

Example 2 – A crystal with pseudo-symmetry 5

Example 3 – SARS-CoV-2 conformers 6

Table S2. $S(C_3)$ for the domains of two SARS-CoV2-spike proteins – all atoms 6

Table S3. $S(C_3)$ for the domains of two SARS-CoV2-spike proteins - backbone. 7

References 7

Connectivity File Format

The connectivity file describes how atoms are connected to each other, and is similar to the CONECT section in the `pdb` file format (https://www.wwpdb.org/documentation/file-format). Atoms are listed sequentially. The first number in each line is an atom serial number, followed by the serial numbers of all the atoms which are chemically bonded to it, regardless of their bond order. As an example, Figure S1 displays the connectivity for a 18C6 molecule without the hydrogen atoms.

```
1 2 18
2 1 3
3 2 4
4 3 5
5 4 6
6 5 7
7 6 8
8 7 9
9 8 10
10 9 11
11 10 12
12 11 13
13 12 14
14 13 15
15 14 16
16 15 17
17 16 18
18 17 1
```

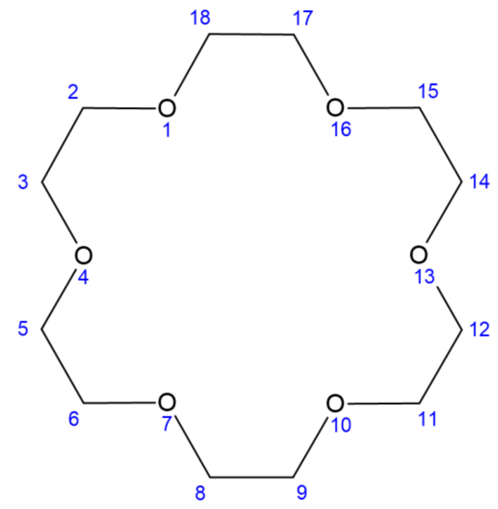

Figure S1: Connectivity file example. The 18C6 molecule without the hydrogen.

The `csm` File Format

The `csm` file format is similar to the `xyz` format with the addition of a connectivity section. The first line is the number of atoms, followed by coordinates section in which separates lines specify the (x, y, z) coordinates of each atom. A connectivity section follows according to the specifications above. Figure S2 exemplifies the `csm` format for a methane molecule.

```
5
C          0.00000        0.00000        0.00000
H          1.09200        0.00000        0.00000
H         -0.36400        0.72800        0.72800
H         -0.36400       -0.99500        0.26700
H         -0.36400        0.26700       -0.99500
1    2    3    4    5
2    1
3    1
4    1
5    1
```

Figure S2: The csm file format example. Methane molecule.

Permutation File Format

Adding a permutation file as input (by means of the `use-perm` flag) forces the CSM algorithm to look for symmetry with respect to the given permutation, and considerably speeds up the calculation. The permutation is provided as a text file with one column listing the atom indices that are to be permuted with the indices 1,2,3,…N respectively, where N is the total number of atoms in the molecule. Table S1 below exemplifies the permutation for reflecting a benzene molecule through two different mirror planes. On the left, the unit permutation is displayed, in which each atom is permuted with itself. This permutation represents a reflection through the horizontal mirror plane that goes through all the atoms in the molecule. On the right column, the permutation represents a reflection through a vertical mirror plane that goes through atoms 2,5,8 and 11.

Table S1: Permutation file format examples. Two different mirror planes of benzene.

**Symmetry Operation**	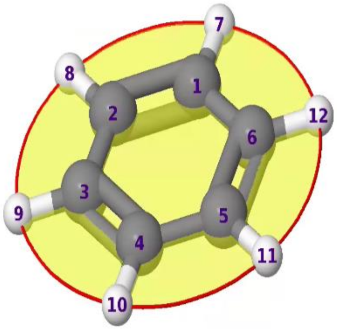	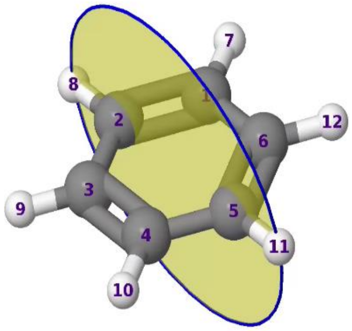
**Permutation**	1<br>2<br>3<br>4<br>5<br>6<br>7<br>8<br>9<br>10<br>11<br>12	3<br>2<br>1<br>6<br>5<br>4<br>9<br>8<br>7<br>12<br>11<br>10

Usage Examples – Run Commands

Below are the command lines for the examples provided in the main text.

Example 1 – Flexible cage molecule

In this example CSM calculations are performed for 18C6 models. Coordinates were extracted from the CSD[1], without the hydrogen atoms or the guest ions, and saved in `sdf` format. For the input and output files see the directory `Example_1` in the file `Examples.zip`.

To run a single calculation on a single file use:

```
csm exact c3 --input input_file_name --output output_dir_name --keep-structure
```

Here `input_file_name` should be replaced with the specific molecular file, including its suffix. The results are written to a directory with the name: `output_dir_name`. The specific name can be chosen by the user. For example, the command line for calculating *S*(C_3) for the file `AWEWOR.sdf` with the output directory `out_awewor` is:

```
csm exact c3 --input AWEWOR.sdf --output out_awewor --keep-structure
```

To run the same command on all the files in the directory replace the `input_file_name` with a dot (.) and change the output directory name as well:

```
csm exact c3 --input . --output out_all_mols --keep-structure
```

Each output file in this case contains the output data of all the runs. Specifically, `csm.txt`, `permutation.txt` and `directional.txt` (the CSM values, final permutation and direction vectors files, respectively) contain a line of results per input file. The two coordinate files (`initial_coordinates` and `resulting_symmetric_coordinates`) contain the molecular geometries as a single concatenated file that can be viewed with an external visualization software such as Jmol.

Note: If the files in the input directory do not have the same format, the `out-format` flag can be used to unify their format in the output file of the nearest symmetry structure. For example:

```
csm exact c3 --input . --output out_all_mols --keep-structure –out-format pdb
```

It is possible to calculate several symmetry measures for one molecule or on all the molecular files in the directory using the `comfile` approach. A command file should be prepared first. The default filename is `cmd.txt` but this is not mandatory. The command file contains the symmetry point group with all the required flags.

For example, the command file (cmd.txt) for calculating $S(C_3)$ and $S(C_2)$ is:

```
exact c3 --keep-structure
exact c2 --keep-structure
```

The run command becomes:

```
csm comfile --input . --output out_c2_c3
```

If the file `cmd.txt` has a different name (e.g., `cmd_file_name.txt`) the run command is:

```
csm comfile cmd_file_name.txt --input . --output out_c2_c3
```

The output files, in this case are more complex:

`csm.txt` becomes a table with a row for each input file and a column for every point group.

`permutation.txt` and `directional.txt` have a result line for every input file and point group.

`The initial_coordinates` file is a concatenated file of all the input structures, regardless of the number of point groups used for the calculation on the same structure.

`The resulting_symmetric_coordinates` file contains a molecular geometry for each combination of initial structure and point group.

Example 2 – A crystal with pseudo-symmetry

The structure was extracted from the CSD[1] and the converted to `sdf` format using Mercury[2], keeping all the atoms of the unit cell. The command line for CSM calculations with respect to the C_i point group is:

```
csm exact ci --input CILHAI.sdf --output out_cilhai --keep-structure
```

For the input and output files see the directory `Example_2` in the file `Examples.zip`.

Example 3 – SARS-CoV-2 conformers

Coordinates for PDB-ID: 7TF8 and 7TO4 were extracted from the RCSB-PDB[3] and cleaned with `pdb_prep`.[4] Given that both input files are at the same directory, the command line for running a CSM calculation is:

```
csm approx c3 --input . --output out_sars_c3 --use-chains --use-sequence
--babel-bond
```

Calculating $S(C_3)$ for the proteins' backbone can be performed using the `–use-backbone` flag:

```
csm approx c3 --input . --output out_backbone --use-chains --use-
sequence –use-backbone --babel-bond
```

Calculating the CSM for the RBD domain is performed by using the `–select-res` flag with the relevant range of residues (330-527):

```
csm approx c3 --input . --output out_rbd --use-chains --use-sequence –
select-res 330-527 --babel-bond
```

Performing the calculations for additional domains can be done with the `comfile` approach as explained above. For the input and output files see the directory `Example_3` in the file `Examples.zip`.

Table S2. $S(C_3)$ for the domains of two SARS-CoV2-spike proteins – all atoms.

**Subunit**	**Domain**	**Residues Number***	**$S(C_3)$ 3-Down PDB-ID = 7TF8**	**$S(C_3)$ 1-Up PDB-ID = 7TO4**
S1	Whole subunit	≤ 685	0.2209	2.5070
	NTD (N-Terminal Domain)	≤ 293	0.2007	0.1213
	N2R (NTD-to-RBD linker)	294-329	0.3406	0.0468
	RBD (Receptor Binding Domain)	330-527	0.3910	12.3141
	SD1 (Sub-Domain 1)	528-590	0.1123	0.0869
	SD2 (Sub-Domain 2)	591-685	0.1984	0.0332
S2	Whole subunit	≥ 686	0.0301	0.0240

* The overall range depends on the experimental data. 7TF8: [27,1147]; 7TO4: [14,1162].

Table S3. ***S*(C_3) for the domains of two SARS-CoV2-spike proteins - backbone.**

Subunit	Domain	Residues Number	*S*(C_3) 3-Down PDB-ID = 7TF8	*S*(C_3) 1-Up PDB-ID = 7TO4
S1	Whole subunit	≤ 685	0.1815	2.4461
S1	NTD (N-Terminal Domain)	≤ 293	0.1813	0.1114
	N2R (NTD-to-RBD linker)	294-329	0.2179	0.0163
	RBD (Receptor Binding Domain)	330-527	0.2812	12.0553
	SD1 (Sub-Domain 1)	528-590	0.0959	0.0501
	SD2 (Sub-Domain 2)	591-685	0.1330	0.0165
S2		≥ 686	0.0163	0.0096

* The overall range depends on the experimental data. 7TF8: [27,1147]; 7TO4: [14,1162].

References

(1) Groom, C. R.; Bruno, I. J.; Lightfoot, M. P.; Ward, S. C. The Cambridge Structural Database. *Acta Cryst.* **2016**, *B72* (2), 171–179.

(2) Macrae, C. F.; Sovago, I.; Cottrell, S. J.; Galek, P. T. A.; McCabe, P.; Pidcock, E.; Platings, M.; Shields, G. P.; Stevens, J. S.; Towler, M.; Wood, P. A. Mercury 4.0: From Visualization to Analysis, Design and Prediction. *J. Appl. Crystallogr.* **2020**, *53* (1), 226–235.

(3) Berman, H. M.; Westbrook, J.; Feng, Z.; Gilliland, G.; Bhat, T. N.; Weissig, H.; Shindyalov, I. N.; Bourne, P. E. The Protein Data Bank. *Nucleic Acids Res.* **2000**, *28* (1), 235–242.

(4) Barhoom, S.; Tuvi-Arad, I. pdb_prep version 0.1.1.0 https://continuous-symmetry-measure.github.io/pdb_prep.
